# Protocol for RNA extraction from glioblastoma cell-line-derived spheroids embedded in atelocollagen gel guided by real-time imaging

**DOI:** 10.1016/j.xpro.2025.103839

**Published:** 2025-05-24

**Authors:** Mayumi Fujita, Kaori Imadome, Tetsuro Sato, Hirokazu Hirakawa, Junko Kado, Asako Yamagiri, Taichi Miura, Satoshi Kamimura

**Affiliations:** 1 Regenerative Therapy Research Group, Department of Radiation Regulatory Science Research, Institutes for Radiological Science, National Institutes for Quantum Science and Technology, Chiba 263-8555, Japan; 2 Product Development Department 3, Koken Co., Ltd., Tokyo 112-0004, Japan

**Keywords:** Cell culture, Cancer, Organoids

## Abstract

Microscopy advancements enable real-time imaging and analysis of cancer spheroids embedded in atelocollagen gel. Here, we present a protocol for extracting high-quality total RNA from 3D tumor spheroids embedded in atelocollagen gel, including spheroids and invading cells dispersed into surrounding gel. We describe steps for spheroid formation, gel embedding, live-cell imaging, and image analysis. We then detail procedures for collecting sufficient quantities of total RNA suitable for comprehensive transcriptome analysis. This approach advances transcriptome profiling in diverse 3D cultured samples.

## Highlights

• This protocol enables time-specific RNA extraction from 3D cancer spheroids in atelocollagen
• Time-specific RNA extraction from 3D cancer spheroids is guided by real-time imaging
• High-quality RNA is obtained from both spheroids and dispersed invading cells
• The method advances transcriptomic profiling in physiologically relevant 3D models

## Abstract (editor highlights)

Publisher’s note: Undertaking any experimental protocol requires adherence to local institutional guidelines for laboratory safety and ethics.

## Graphical abstract

**Figure:**
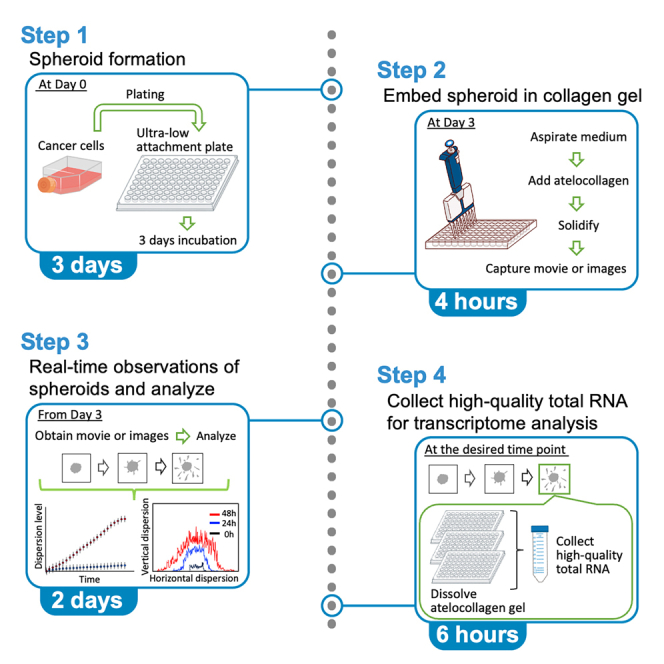

## Before you begin

This protocol describes a method for collecting a sufficient amount of high-quality total RNA samples by rapidly dissolving spheroids and dispersed cells from the spheroid into atelocollagen gel (The invaded cells) at a desired time point as determined by real-time observation and analysis. However, this protocol can also be applied for various 3D culture samples embedded in atelocollagen gel. In addition, atelocollagen is known as a low-immunogenic type I collagen derivative and is clinically applied in wound healing, bone cartilage substitutes, and hemostasis.^1^ Thus, we used atelocollagen in this protocol development, with a view to its use in basic research for future clinical applications. The procedure consists of four major steps: (1) Generate 3D Spheroids; Use cancer cells to generate 3D spheroids in an ultra-low attachment (ULA) 96-well plate. (2) Embed the Spheroids in Atelocollagen Gel, (3) monitor the spheroid and Analyze; Start live cell imaging to monitor the spheroid as well as the invading cells moving away from the spheroid into the atelocollagen gel. Analyze the degree of dispersion of invading cells from the spheroid. (4) Collect a large amount of High-quality Total RNA; at the desired sampling point identified during real-time imaging and its analysis, rapidly dissolve the spheroids and invaded cells to collect total RNA. We will also present an example of the results of comprehensive gene expression analysis using this total RNA. We have optimized the assay for the glioblastoma multiforme cell line, SF126.

### Preparation of the atelocollagen solution stocks for embedding cancer spheroids

**Timing: 2 days**

***Note:*** Keep the atelocollagen solution on ice during dispensing to maintain its temperature. If the solution temperature rises, the collagen will fibrillate and form a gel. All steps that will come in contact with cells must be performed inside a clean bench.

1. Aliquot atelocollagen gel.
  Day 1.
  a. Thaw atelocollagen solutions (3D Ready Atelocollagen-DMEM low glucose, 4 mg/mL, pH 7.4, Koken) for 8 to 12 h by placing the bottle in a refrigerator.
  b. Acquire materials needed to dispense the ice-cold atelocollagen solution such as sterile 1.5 mL tubes, sterile pipette tips (200 μL, and 1000 μL), and tube racks.
    Day 2.
  c. Fill the bucket with ice, and place a tube rack on the ice.
  d. Place the 1.5 mL tubes by placing into the tube rack.
  e. Place the bottle containing atelocollagen solution on the ice and swirl the bottle to make sure that atelocollagen solution is evenly dispersed.
  f. Open the bottle on the ice, and use the pipette tips to aliquot the atelocollagen solution (500 μL) into the cold 1.5 mL tubes.
  g. Label the tubes, and store them at −20°C for up to one year.

### Preparation of the cancer cell line, SF126

**Timing: 3 days**

This step describes the preparation of cancer cells, SF126, to use for the spheroid formation. All steps that will come in contact with cells must be performed inside a clean bench.

2. Preparation of the cancer cell line, SF126.
  a. Thaw a frozen stock of SF126 (1.25 × 10^6^ cells).
  b. Suspend SF126 in 10 mL DMEM-low glucose supplemented with 10% fetal bovine serum (FBS) and 1% penicillin streptomycin.
  c. Centrifuge SF126 with 150 × g for 5 min, resuspend cells with the fresh 10 mL DMEM, and plate it into 100 mm culture dish.
  d. Incubate SF126 at 37°C, 5% CO2 for 3 days.

***Note:*** 1.25 × 10^6^ SF126 cells per 100 mm culture dish can grow to approximately 5 - 6 × 10^6^ (about 80 to 90% confluency after 3 days incubation.

## Key resources table

**Table:** 

REAGENT or RESOURCE	SOURCE	IDENTIFIER
**Chemicals, peptides, and recombinant proteins**		

3D Ready Atelocollagen DMEM-LG1 (low glucose)	Koken	3D-LG01
DMEM, low glucose, GlutaMAX supplement, pyruvate	Gibco	10567014
Dulbecco’s phosphate-buffered saline (PBS)	Nacalai Tesque	14249–24
Fetal bovine serum (FBS)	Sigma	F173012
Penicillin-streptomycin	Nacalai Tesque	26252–94
0.5% Trypsin-EDTA	Gibco	15400054
0.05% Trypan blue solution	Nacalai Tesque	20577–34
2-Mercaptoethanol (β-mercaptoethanol)	Sigma	M3148-250ML
Ethanol (99.5)	FUJIFILM Wako	057–00456

**Critical commercial assays**		

RNeasy mini kit	QIAGEN	74104

**Experimental models: Cell lines**		

SF126	https://web.expasy.org/cellosaurus/CVCL_1688	RRID:CVCL_1688
MIA PaCa-2	https://web.expasy.org/cellosaurus/CVCL_0428	RRID:CVCL_0428

**Oligonucleotides**		

Primer: CDK1 Forward: AAACTACAGGTCAAGTGGTAGCC Reverse: TCCTGCATAAGCACATCCTGA	Primer Bank	PrimerBank ID 281427275c1
Primer: CCNB1 Forward: AATAAGGCGAAGATCAACATGGC Reverse: TTTGTTACCAATGTCCCCAAGAG	Primer Bank	PrimerBank ID 356582356c1
Primer: CDC25C Forward: ATGACAATGGAAACTTGGTGGAC Reverse: GGAGCGATATAGGCCACTTCTG	Primer Bank	PrimerBank ID 125625348c2
Primer: AULKA Forward: GAGGTCCAAAACGTGTTCTCG Reverse: ACAGGATGAGGTACACTGGTTG	Primer Bank	PrimerBank ID 38327563c1
Primer: CXCL8 Forward: ACTGAGAGTGATTGAGAGTGGAC Reverse: AACCCTCTGCACCCAGTTTTC	Primer Bank	PrimerBank ID 10834978a2
Primer: IL1B Forward: AGCTACGAATCTCCGACCAC Reverse: CGTTATCCCATGTGTCGAAGAA	Primer Bank	PrimerBank ID 27894305c2
Primer: SOD2 Forward: GCTCCGGTTTTGGGGTATCTG Reverse: GCGTTGATGTGAGGTTCCAG	Primer Bank	PrimerBank ID 67782304c1

**Software and algorithms**		

ImageJ Fiji	https://imagej.net/	RRID: SCR_003070
INSIDIA	https://valentinapalmieri.wixsite.com/insidia	N/A

**Other**		

1.5 mL tube (autoclavable, RNase free)	TreffLab	96.08668.9.01
Sterile 200 μL pipette tips (filtered)	Thermo Fisher Scientific	2770PK
Sterile 1000 μL pipette tips (filtered)	Thermo Fisher Scientific	2279PK
10 mm TC-treated cell culture dish	Falcon	353003
Conical tubes: 15 mL	Falcon	352196
Conical tubes: 50 mL	Falcon	352070
5 mL pipette (filtered)	Falcon	357543
10 mL pipette (filtered)	Falcon	357551
Disposable cell counter	Waken BTech Co., Ltd.	WC2-100
96-well clear round bottom ultra-low attachment (ULA) microplate	Corning	7007
Inverted microscope	Olympus	CK40
IncuCyte Zoom live-cell analysis system	Sartorius (formerly, Essen BioScience)	IncuCyte ZOOM
20 mL syringe	Terumo	SS-20ES2
20-gauge needle	Terumo	NN-2038R
Nanodrop spectrophotometer	Thermo Fisher Scientific	ND3300LAPTOP

## Step-by-step method details

### Part 1: Create cancer spheroid

#### Preparation of the cancer spheroid using SF126 cell line

**Timing: 3 days**

This step describes the method of spheroid formation with SF126 for three ULA 96-well plates; one plate for the n=1 control sample (the sample of spheroids that are not embedded in atelocollagen gel) and the remaining two plates for the n=1 test sample (the sample of spheroids that are embedded in atelocollagen gel).

***Note:*** To collect the enough amount of the total RNA to use for the comprehensive gene expression analysis via dissolving the atelocollagen gel (the test sample), spheroids collected from at least two plates are needed to make n=1 sample. If you are going to prepare n=3 samples for each group, you will need 3 plates (1 plate x 3 sets) for the control group, and 6 plates (2 plates x 3 sets) for the atelocollagen-embedded group. All steps that will come in contact with cells must be performed inside a clean bench.

**Figure 1:**
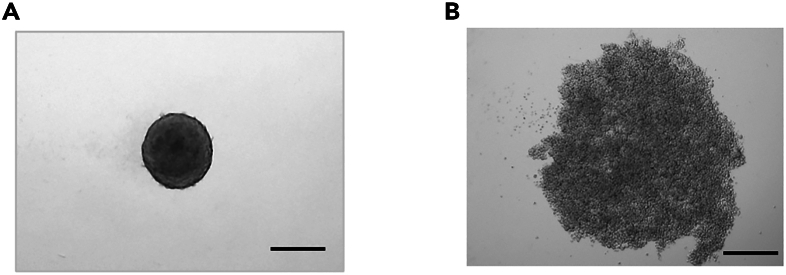
Image of spheroid with compact spherical structure (A) Human glioblastoma multiforme cell line (SF126), or (B) human pancreatic cancer cell line (Miapaca-2) with 8 × 10^3^ cells were plated in each well of ULA 96-well plate, and incubated at 37°C, 5% CO2 for 3 days to generate the spheroid. Representative image of spheroid photographed with a BZ-8000 microscope (Keyence, Osaka, Japan) using a 4× PlanApo lens (N.A 0.20). Scale bar: 300 μm.

1. Formation of spheroid with using SF126.
  Day 1.
  a. Check SF126 plated in 100 mm culture dish to make sure that the cells are in logarithmic growth phase with appropriate density, about 70 to 85% confluency.
  b. Remove DMEM medium from the culture dish by gentle aspiration.
  c. Wash the cells with 10 mL sterile pre-warmed (37°C) 1 × PBS.
  d. Add 1 mL of 0.5% Trypsin-EDTA, and make sure that cells are all covered.
  e. Incubate the dish in 5% CO_2_ incubator at 37°C for 2 min.
  f. Check the cells under the optical microscope, and make sure most of the cells are detached from the dish.
  g. Add 5 mL of DMEM medium to stop the trypsin-EDTA reaction.
  h. Collect all the cells from the plate in a 15 mL conical tube.
  i. Take 50 μL of cell solution and mix well with the same amount of trypan blue solution, and count the live cell number with using disposable cell counter.
  j. Centrifuge the cell solution in 15 mL conical tube for 5 min at 150 × g at room temperature (25°C).
  k. Remove the medium, and resuspend the cells to be the concentration of 8 × 10^4^ cells/mL with fresh DMEM medium.
  l. Dispense 100 μL of cell solution (8 × 10^4^ cells/mL) into each well of three ULA 96-well plates. (Each well contains 8 × 10^3^ SF126 cells.)
  m. Incubate ULA plates at 37°C, 5% CO2 for 3 days to generate the spheroid.
    Day 3.
  n. Check the spheroid with the optimal microscope, and make sure each spheroid in each well forms compact spherical structure (Figure 1A).
    ***Note:*** Some cell lines may not be able to form compact spherical spheroids (Figure 1B). In such cases, embedding them in atelocollagen gel will cause their structure to disperse even further, so such cell lines are not suitable for use in the assay to evaluate the dispersion level of cancer cell invasion from the spheroid. However, as long as the cell lines can form compact spheroids on ULA plates and remain intact when embedded in atelocollagen gel, they would be suitable for further evaluation.

### Part 2: Embed spheroid in atelocollagen gel

#### Embedding SF126 spheroid in the atelocollagen gel

**Timing: 4 h**

This step describes the embedding SF126 spheroid in the atelocollagen gel. All steps that will come in contact with cells must be performed inside a clean bench.

***Note:*** Thaw four tubes of the atelocollagen solution aliquot (500 μL/tube) by placing them in a refrigerator at 4°C for 8 to 12 h prior to Day 3. Atelocollagen solutions should be handled on ice during preparation. Pre-cooling of materials, sterile pipette tips (20 μL, 200 μL, and 1000 μL), and tube racks is not required. Also, prepare the ice-cold FBS by placing them in a refrigerator.

2. Preparation of atelocollagen gel for embedding the spheroid.
  Day 3 - continued.
  a. Fill the bucket with ice and place a tube rack on the ice.
  b. Place the four tubes containing the atelocollagen solution (500 μL/tube), which have been thawed in the refrigerator since the day before, in a tube rack.
  c. Add 5 μL of ice-cold FBS into each 500 μL atelocollagen solution to make the atelocollagen solution supplemented with 1% FBS.
  d. Mix the atelocollagen solution well using a 1000 μL pipette tip.
    **CRITICAL:** Because 4 mg/mL (0.4%) atelocollagen solution is a highly viscous solution that easily contains air bubbles, we highly recommend mixing the solution slowly and carefully. If you embed the spheroid within atelocollagen gel that contains air bubbles, you will not be able to get clear images when observing the cells that subsequently invade from the spheroid into the atelocollagen gel.
3. Embed cancer spheroid in atelocollagen gel.
  a. Place the ULA 96-well plate containing spheroids on ice, and carefully remove 75 μL medium from each well with using 200 μL pipette. Be sure not to aspirate the spheroid while aspirating the medium.
  b. Use 200 μL pipette tips, and slowly add 75 μL of ice-cold atelocollagen solution into each well.
    **CRITICAL:** We recommend pipetting the atelocollagen solution for few times and eject the bubbles before adding the solution into the well.
  c. Repeat step a and b for two ULA plates (The test sample). The one ULA plate left is used as the plate for the control sample (the spheroids without embedded in the atelocollagen gel).
  d. Incubate the atelocollagen solution in ULA plates in CO_2_ incubator at 37°C with 5% CO_2_ for 2 h to form a gel.
    Option: Chemicals of your interest, such as inhibitors, can be added to the atelocollagen solutions. For example, you can screen for inhibitors that are effective in suppressing cell invasion from the cancer spheroids. You can also harvest the cells and perform a comprehensive gene expression analysis to see the effect of the chemicals on the cells. However, we recommend that you use atelocollagen gel at a concentration higher than 2 mg/mL, even if you mix it with the chemicals. If spheroids are embedded in atelocollagen gel at a concentration of less than 2 mg/mL, the gel will not harden enough, and the spheroids may move slightly within the gel without being fixed in place. This may cause the focus to shift during real-time imaging, making it impossible to obtain a clear image.

### Part 3: Capture live-cell images and analysis

#### Image acquisition of the live-cell imaging and their analysis

**Timing: from day 3 until the desired sampling point (for example: 2 days)**

This step describes the method of using the Incucyte Zoom Live-Cell Analysis System to obtain live cell imaging movie or time-course images to kinetically monitor spheroid changes.

***Note:*** Cancer cell invasion from the spheroid into the atelocollagen gel can be monitored by the any of the live-cell imaging system such as Incucyte Zoom Live-Cell Analysis System (Sartorius, Göttingen, Germany) or BZ-X810 microscope with Time-lapse module (Keyence, Osaka, Japan).

**Figure 2:**
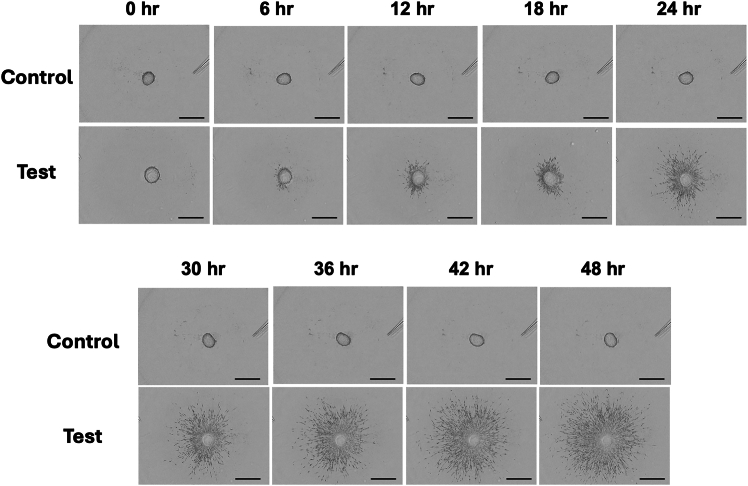
Live cell imaging of spheroid embedded in collagen gel and its analysis Live cell imaging of the control sample (spheroids that are not embedded in collagen gel) and the test sample (spheroids that are embedded in collagen gel) were performed using Incucyte Zoom Live-Cell Analysis System (Sartorius) with spheroid analysis software. Phase-contrast images of spheroid every 1 h were used to create movie (Methods video S1). Representative tiff image of every 6 h taken from the movie are shown. Each image shows scale bar at the left bottom (800 μm), and the elapsed time at the right bottom.
  c. Quantify the dispersion level of invaded cells from the spheroid into atelocollagen gel overtime with using the Incucyte zoom spheroid analysis software.
    ***Note:*** If you do not have such application, phase-contrast tiff images with the desired time course can be used and quantify the dispersion level of the invaded cells using INSIDIA (INvasion SpheroID ImageJ Analysis)^2^ as described in the next section.

4. Live cell imaging and their analysis.
  a. Set both of the control ULA plates, containing the spheroid without embedded in atelocollagen gel, or the test plates, having the spheroid embedded in atelocollagen gel, into the Incucyte Zoom Live-cell system.
  b. Photograph the spheroid automatically with your desired time intervals such as at 1 h intervals using a 4× objective until the time you want to collect the total RNA.
    ***Note:*** Movie or time-course images to kinetically monitor spheroid changes can be created with the phase-contrast images that has been taken for example with every 1 h (Methods video S1; movie for the control spheroid, Methods video S2; movie for the test spheroid, Figure 2; the tiff images of every 6 h taken from the movie).

Methods video S1. Time-lapse movie of control spheroid (non-embedded in atelocollagen gel)

Methods video S2. Time-lapse movie of test spheroid (embedded in atelocollagen gel)

#### Quantify the dispersion level of invaded cells with ImageJ Fiji using INSIDIA open-source macro

**Timing: 3 h**

This step describes the optimized protocol of analyzing the dispersion level of invaded cells from the spheroid by using INSIDIA, an open-source macro for the Fiji platform ImageJ software (Figure 3).

5. Preparation of INSIDIA on your PC.
  a. Download ImageJ Fiji (https://imagej.net/software/fiji/downloads)^2^ onto your PC.
  b. Prepare the open-source macro file, INSIDIA, as downloading ‘INSIDIA-2022.txt’ (Figure 3A), and INSIDIA sample files (https://valentinapalmieri.wixsite.com/insidia).^3^ The user manual ‘INSIDIA GUIDE’ is also available at (https://valentinapalmieri.wixsite.com/insidia/quick-user-guide).
  c. Open Fiji, click ‘File’, select ‘New’ and then select ‘Script’ (Figure 3B).
  d. Open the file of ‘INSIDIA-2022.txt’ and copy all of the contents.
  e. Paste the contents into the ‘Scripts’ window of Fiji (Figure 3C).
  f. Select Fiji, click ‘Language’, and choose ‘ImageJ Macro’, then INSIDIA is ready to use.
6. Preparation of the spheroid segmented image using the INSIDIA application.
  a. Open ‘Experiment 0’ folder already stored in the ‘Main folder’ of INSIDIA application and store the tiff images for the analysis (Figure 3D).
  b. Click ‘Run’ at the bottom left of the Fiji script window (Figure 3C), and select ‘Main folder’ and click ‘Open’ (Figure 3E), then the popup window comes up (Figure 3F).
  c. Select ‘No, I want to do spheroid segmentation’, and click ‘OK’, then the spheroid segmentation of the images that you saved in the Main folder will automatically be proceeded (Figure 3G).
    **CRITICAL:** This automatic spheroid segmentation works very well with spheroid images that have a low background and clear spheroid outlines; the yellow line shows the automatically selected spheroid (Figure 3G). On the other hand, this segmentation is hard to be used for the spheroid with dispersed invasion cells; the automatically selected yellow line shows the edge of the image, not the spheroid with invaded cells (Figure 3H). In that case, you can use the method of next step, step d.
  d. In case of your image failed the automatic spheroid segmentation, you alternatively can use the ‘manual threshold’ and set the threshold by your own (Figure 3I).
  e. Select ‘Dark background’ of the threshold panel and click ‘Apply’, then you can get the ‘mask image’ (Figure 3I).
  f. Select Fiji, click ‘file’, and choose ‘Save as’ to save the ‘mask image’ as tiff file. Proceed the step d to f for preparing all mask images you want to analyze.
    **CRITICAL:** In order to accurately compare all images being analyzed, the conditions used for spheroid segmentation must be the same for all images. For example, if you are using manual segmentation, proceed manual thresholding on all images, including images of the control samples, and be sure to use the same threshold and prepare the mask image.
7. Preparation for image quantification in Fiji application using international system unit rather than pixels.
  a. Open Fiji application and open an image containing the scale (Figure 4A).

**Figure 4:**
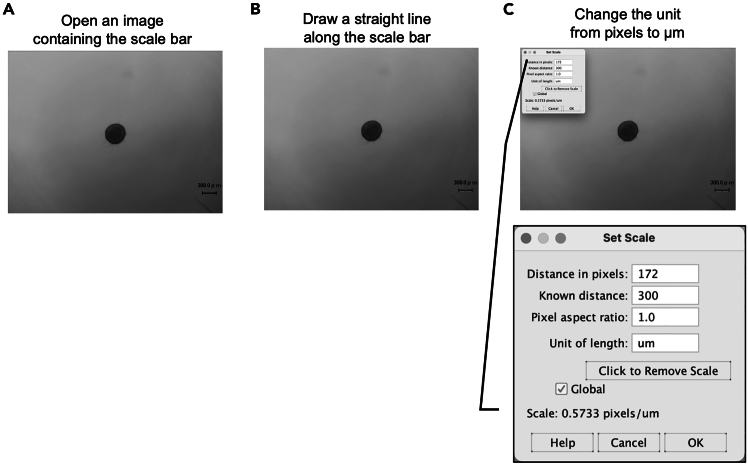
Preparation for image quantification in Fiji using international system unit rather than pixels (A) Prepare an image containing the scale bar. (B) Use ‘Line tool’ of Fiji application and draw a straight line along the scale bar. (C) In the dialog of ‘Set Scale’, the unit can be changed from pixels to an international system unit, such as micrometers (μm).
  b. Use ‘Line tool’ in Fiji to draw a straight line along the scale bar on the image (Figure 4B).
  c. Click on ‘Analyze’ and select ‘Set Scale’. In the dialog that appears, you can change the unit from pixels to an international system unit, such as micrometers (μm) (Figure 4C).
  d. Check the ‘Global’ box in the dialog (Figure 4C). This will apply the selected unit (e.g., μm) to all subsequent image quantification steps.
8. Quantify the dispersion level of invaded cells using Fiji application.
  a. Open Fiji application and then open the black-and-white ‘mask image’ created with the INSIDIA open-source macro.
  b. Press Ctrl+A to select whole area of the ‘mask image’.
  c. Click ‘Analyze’ and select ‘Plot Profile’, then the vertical sum of the grayscale intensity for the image pixels is plotted to the graph (Figure 5).

**Figure 5:**
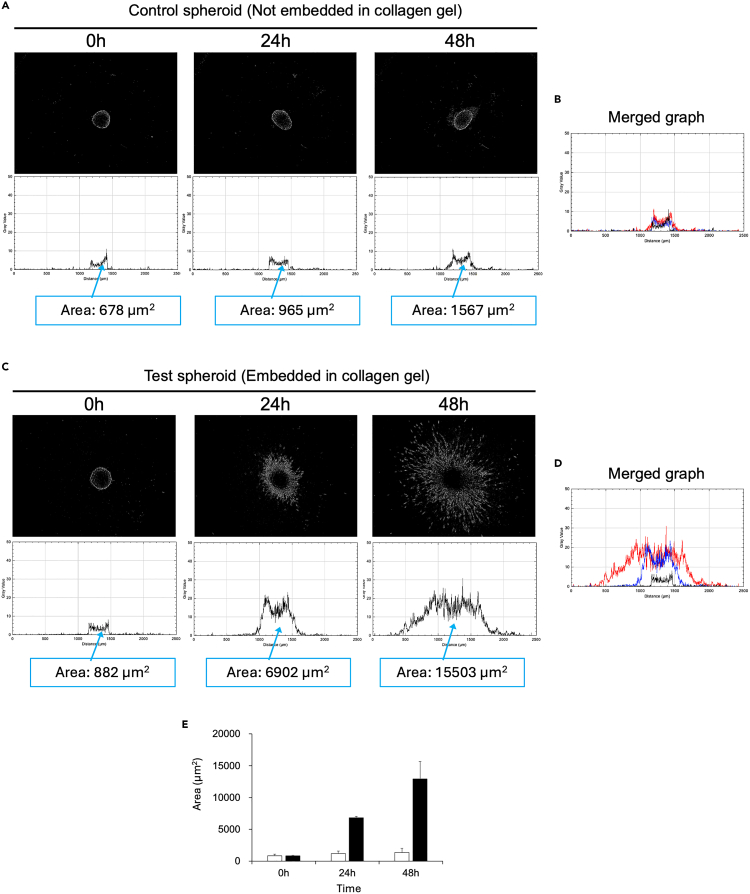
Quantification of the dispersion level of invaded cells using Fiji application Create graph for the ‘mask image’ with using the ‘plot profile’ Fiji application and measure the plot area using ‘wand tool’. The dispersion levels of invaded cells from the spheroid were evaluated via quantifying each plot area. (A) Representative results of the time-course changes of the spheroid morphology were shown using the control sample images. (B) The time-course results of the control sample can be merged in one graph. (C) Representative results of the time-course changes of the dispersion levels of invaded cells from the spheroid of the test sample were shown. (D) The time-course results of the test samples can be merged in one graph.
  d. Perform the steps of a-c for all the ‘mask images’ you want to analyze and create the intensity plot of each ‘mask image’.
  e. Use ‘Wand tool’ of Fiji and measure the area of each plot (Figures 5A and 5C), then you can get total dispersed level of spheroid from each image (Figures 5A and 5C).

**Figure 3:**
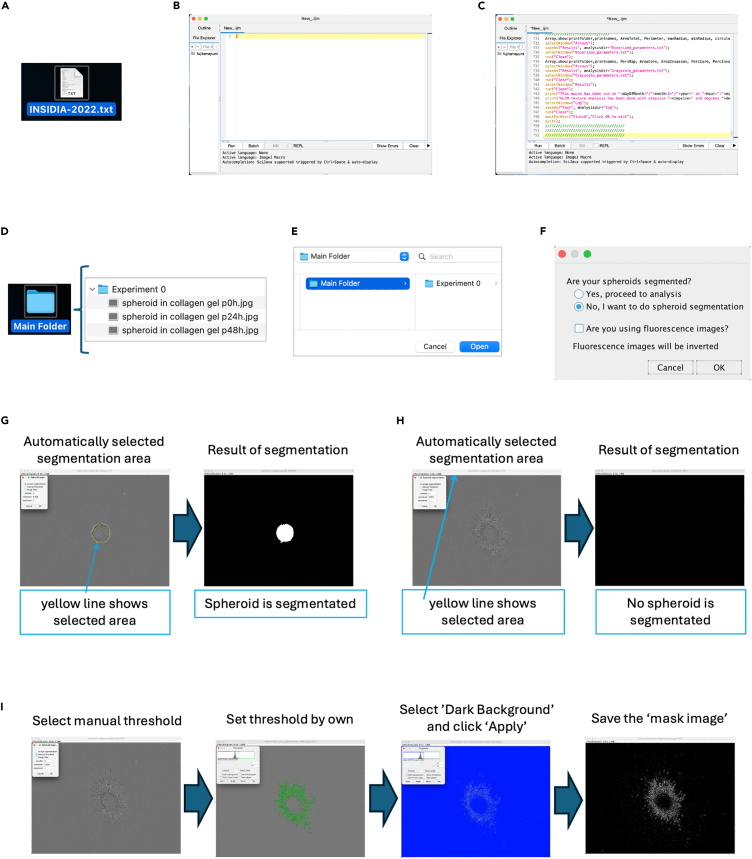
Method for the image segmentation of spheroid and dispersed invaded cells using the INSIDIA open-source macro (A) Download the open-source macro file, INSIDIA-2022.txt, on your PC. (B) Download the ImageJ Fiji application and open the script window. (C) Copy the text of the INSIDIA-2022 file and paste into the script window of Fiji. (D) Save the images that you want of analyze in the Experiment Folder stored in the Main Folder. (E) Run the script of Fiji and select the Main Folder. (F) Select ‘No’ in the popup window, and then the image segmentation of spheroid and dispersed invaded cells will start automatically. (G) Example of successful automatic image segmentation. (H) Example of unsuccessful automatic image segmentation. (I) Method to manually proceed the image segmentation by stating threshold to select the spheroid and dispersed invaded cells.

Option: You can also overlay multiple graphs as clicking ‘Data’ shown in the lower left corner of the plot, and selecting ‘add from plot’. Then, the time-course dispersion of invading cells can be shown in the graph (Figures 5B and 5D).

***Note:*** As the background of the ‘mask image’ is black color, the outline of the spheroid and the invaded cells that have dispersed from the spheroid are shown in white color. By calculating the vertical sum of the grayscale intensity of each pixel making up the image and plotted into the graph, sum of the dispersion level of invaded cells from the spheroid can be shown in a graph (Figures 5A and 5C). The area of the graph indicates the total dispersion level of the invaded cells in the horizontal and vertical directions (Figures 5B and 5D) so that you can compare the dispersion levels of spheroid of each ‘mask image’ by calculating this area (Figure 5E).

***Note:*** This protocol can also be applied for the analyzing simple spheroid growth or the any of your 3D cultured samples embedded in atelocollagen gel.

### Part 4: Collect an enough amount of high-quality total RNA for comprehensive gene expression analysis

#### Quickly collecting high-quality total RNA from the whole atelocollagen gels containing the spheroid and dispersed invaded cells

**Timing: 6 h (you can temporally stop the experiment at the end of step 9 by storing samples at −80°C. In that case, the timing would be about 2 h)**

This step describes the method of rapidly and easily sampling the atelocollagen gel containing the spheroid and dispersed invaded cells from the each well of the ULA plate, and dissolving them to collect the total RNA. We optimized a protocol with using RNeasy mini kit (QIAGEN) to collect high-quality total RNA.

**CRITICAL:** Any item that will come in contact with the spheroid samples should be RNase-free including tubes and tips.

***Note:*** Prepare RLT buffer with adding β-Mercaptoethanol (10 μl β-ME/1 mL RLT buffer) (β-ME is regulated as a hazardous chemical worldwide, so the use of experiments and their disposal methods should be conducted in compliance with the established regulations of each country) prior to the day of sampling (The RLT containing β-ME can be stored at room temperature at 25°C for up to one month). Also prepare RPE buffer by adding four times the volume of 100% EtOH to Buffer RPE. Set the micro centrifuge to room temperature (25°C).

9. Collect the atelocollagen gels containing the 3D spheroid and invaded cells from ULA plate and lysis them.
  a. For the test plates (two ULA plates containing the spheroid embedded in atelocollagen gel), place a Kim towel on the lab bench.
  b. Hold the ULA plate firmly with your hand so that the lid does not open, and place it on the Kim towel with the lid facing down (Figure 6A).
    ***Note:*** At this point, you can see that the pink-colored atelocollagen gel is attached to the bottom of each well of the ULA plate.

**Figure 6:**
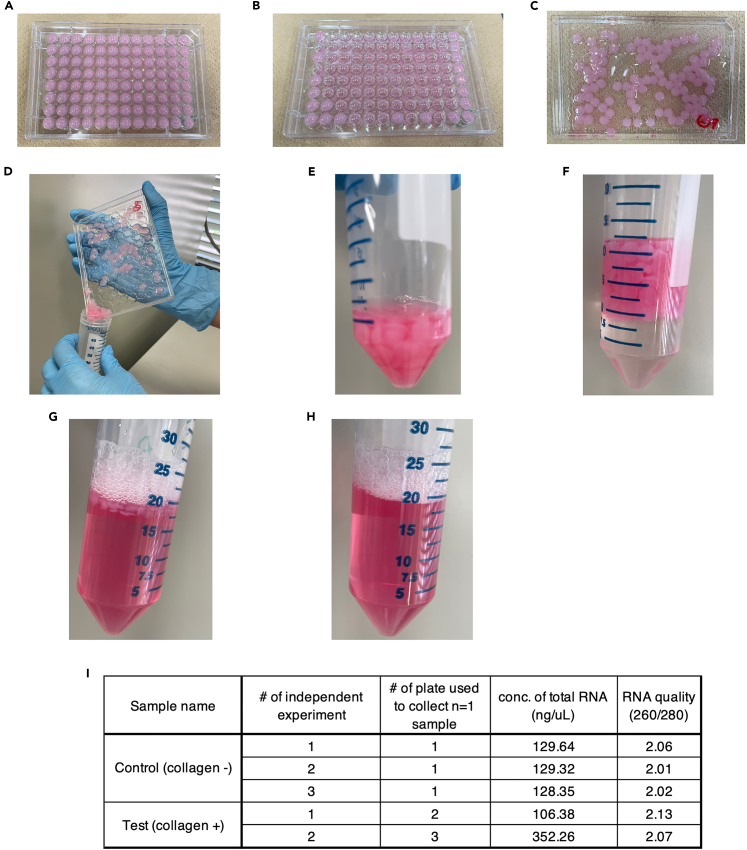
Steps for collecting high-quality total RNA from the whole collagen gels containing the spheroid and dispersed invaded cells (A) Place the ULA plate containing the spheroid embedded in collagen gel on the Kim towel with the lid facing down. (B) Hold the ULA plate firmly and strike the plate against the Kim towel for five times. Photo represents the image after three times striking. (C) Collect the collagen gels on the lid by slowly lift up the main body of the plate. (D) Carefully transfer the collagen gel from the lid into the 50 mL tube. (E) Centrifuge the 50 mL conical tube for 5 min at 150 × g and discard the medium. (F) Add the same amount of RLT buffer as the collagen gel solution to the tube. (G) Photo of the lysate solution 2 min after vigorous mixing. (H) Photo of the lysate solution 3 min after vigorous mixing. The lysate is then used to extract high-quality total RNA by using Rneasy mini kit. (I) Total RNA concentration and their quality collected from the several independent experiments using the method above were shown.
  c. While holding the ULA plate firmly so that the lid does not open, strike the plate against the Kim towel for five times.
    ***Note:*** You can see the atelocollagen gel that was stuck to the bottom of each well of the ULA plate has peeled off from the well and fallen onto the lid (the bottom of the well becomes transparent) (Figure 6B). Make sure that the atelocollagen gel has peeled off from all the wells.
  d. Leave only the plate lid on the Kim wipe, and slowly lift up the main body of the plate.
    ***Note:*** The atelocollagen gels that have peeled off from the wells will be collected on the lid (Figure 6C).
  e. Check that there is no atelocollagen gel left inside the wells (Most of the gels should have fallen onto the lid after five times striking).
    ***Note:*** In case if any gel remains in the well, use the tip of the 200 μl chip to pick up the gel and drop it onto the lid.
  f. Open the lid of the 50 mL conical tube, place the corner of the plate lid on the tube, and slowly tilt the lid at an angle to transfer the atelocollagen gel into the 50 mL tube (Figure 6D).
    ***Note:*** The atelocollagen gel will slide smoothly into the 50 mL tube along with the medium, so you can easily collect every gel into the tube.
  g. Proceed steps a to e for the two plates, and mix the collected atelocollagen gel together to make a n=1 test sample.
  h. Centrifuge the 50 mL conical tube containing atelocollagen gel solution for 5 min at 150 × g at room temperature (25°C) and discard the medium as much as possible (Figure 6E).
    ***Note:*** The amount of the collected atelocollagen gel would be approximately 9 mL.
  i. Add the same amount of RLT buffer (9 mL) as the atelocollagen gel solution to the tube (Figure 6F).
  j. Tightly close the lid of the 50 mL tube, shake the tube up and down vigorously to mix the liquid, and dissolve the atelocollagen gel, spheroid, and invaded cells.
    **CRITICAL:**Figure 6G shows the lysate solution photographed 2 min after vigorous mixing (some gels are not dissociated yet and still remaining in the solution). In most cases, the atelocollagen gel will be completely dissolved by 3 min (Figure 6H). Even if the atelocollagen gel has dissolved and is no longer visible, you may still be able to see the white spheroid particles. In this case, shake the tube up and down vigorously for further 3-5 min to further dissolve the remaining spheroid.
  k. Homogenize the lysates using a 20 mL syringe with a 20-gauge needle by drawing the lysate in and out for 10 times.
    OPTION: The QIAshredder homogenizer (QIAGEN) can be used to replace syringe-and-needle homogenization.
  l. Centrifuge the 50 mL conical tube for 5 min at 150 × g at room temperature (25°C) and collect the sup into the new 50 mL conical tube.
    ***Note:*** The lysate can be stored at −80°C for up to few months.
  m. For the control samples (the ULA plates without atelocollagen gels), use the wide 200 μl tip to collect every spheroid from all the wells and collect them into the 50 mL conical tubes. Then, repeat step g to k to prepare the n=1 control sample.
10. Collect the high-quality total RNA from the spheroid lysate.
  a. Prepare the RNeasy kit (QIAGEN).
  b. Add approximately the same amount of 70% EtOH to the lysate solution (about 18 mL), and mix well together. The lysate solution will be about 36 mL.
  c. Set the RNeasy Mini Spin Column into a 2 ml collection tube.
    ***Note:*** Prepare four set of this; two columns are for a control sample, and rest of two columns are for a test sample.
    **CRITICAL:** Only 700 μL of lysate can be loaded into one column at a time. As there are 36 mL of lysate solution, it would take approximately 51 centrifuges to process 36 mL lysate in one column, which would take a lot of work. So, instead of using one column for 36 mL of lysate, two columns are better to be used at the same time.
  d. Apply approximately 700 μL of lysate into each column.
  e. Centrifuge column at 14,000 × g for 15 s, and discard the flow-through in the collection tube.
  f. Reset the mini spin column into the collection tube and repeat the step d and e until all the lysate will be used. (Approximately 25 to 26 times of centrifuge will be needed.)
  g. Apply 700 μL of Buffer RW1 to each column and centrifuge at 14,000 × g for 15 s.
  h. Set the mini spin column into a new 2 ml collection tube.
  i. Apply 500 μL of Buffer RPE to the column and centrifuge at 14,000 × g for 15 s.
  j. Discard the flow-through in the collection tube and reseat the column.
  k. Apply 500 μl of Buffer RPE to the column and centrifuge at 14,000 × g for 2 min to completely remove the carryover of Buffer RPE.
  l. Set the column in a new 2 ml collection tube and centrifuge at 14,000 × g for 1 min.
  m. Set the column in a new 1.5 ml collection tube and add 34 μl of RNase-free water into only the first column.
  n. Centrifuge at 14,000 × g for 1 min to elute the RNA. Repeat this centrifuge twice.
  o. Collect RNA elution from the first column and add into the second column.
  p. Centrifuge the second column at 14,000 × g for 1 min to elute the RNA. Repeat this centrifuge twice.
    **CRITICAL:** To collect high concentration of total RNA from n=1 sample, the solution eluted through the first column with 34 μL of water is better to be collected, and use it for the second column elution. As approximately 2 μL of solution is lost in each elution, the final RNA solution will be approximately 30 μL.
  q. Measure the OD value using a spectrophotometer. The enough amount of high-quality total RNA for comprehensive gene expression is obtained (Figure 6I).
    ***Note:*** Comprehensive gene expression analysis such as GeneChip Clariom S Assay (Filgen, Inc., Aichi, Japan), for example, requires at least 1 μg of total RNA at a concentration of higher than 100 ng/μL.
    ***Note:*** As an example of results obtained using the GeneChip Clariom S Assay analyzing total RNA prepared by us, we present some of the results; comparison of mRNA expression levels between two samples: a control sample (spheroids not embedded in atelocollagen gel) and a test sample (spheroids embedded in atelocollagen gel). Expression levels of transcripts were compared between control and test samples, and those more than 2-fold down-regulated or more than 2-fold up-regulated in test sample vs. control sample were shown in Tables S1 and S2, respectively. We then used the gene lists shown in Tables S1 and S2 and performed gene ontology (GO) analysis. Figure 7A shows the top 5 biological processes detected in the GO analysis; down-regulation of cell cycle and up-regulation of tumor necrosis factor response or cell migration are detected as the significant biological processes in the test sample compared to the control sample. For the validation study, we further prepared n=3 samples for each control or test group, and performed the RT-qPCR for the genes related to cell cycle (Figure 7B) and tumor necrosis factor response or cell migration (Figure 7C).

**Figure 7:**
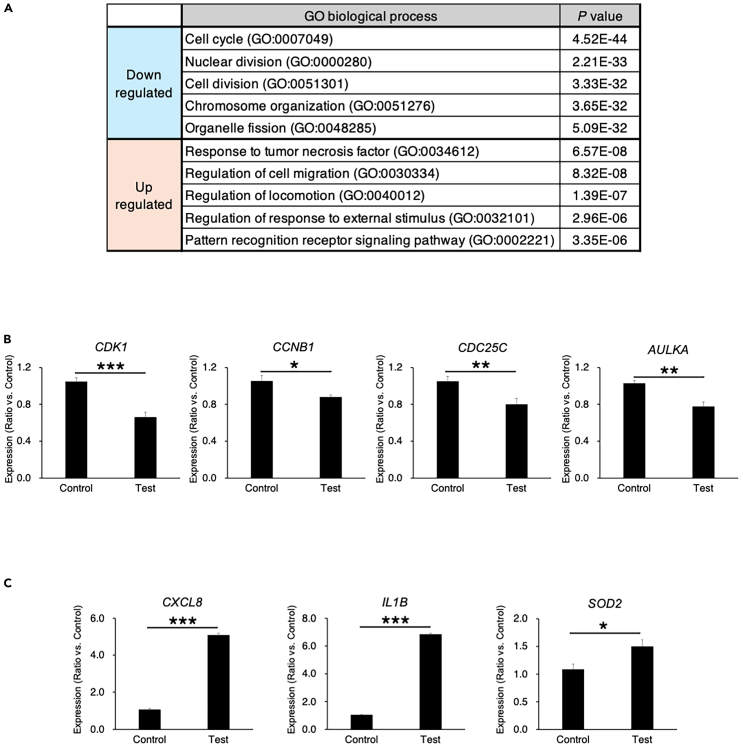
Results of comprehensive gene expression analysis using total RNA prepared from the collagen gel containing SF126 spheroids and their invaded cells (A) The GeneChip Clariom S Assay was performed with using total RNA of a control sample (spheroids not embedded in collagen gel) and a test sample (spheroids embedded in collagen gel). (n=1) Expression levels of transcripts between control and test samples were compared and those more than 2 fold down-regulated (Table S1), or more than 2 fold up-regulated (Table S2) in test sample vs control sample were used for the gene ontology (GO) analysis. Top 5 biological process detected in GO analysis are listed in the table. RT-qPCR verification was performed for the genes related to the cell cycle (B), and tumor necrosis factor response or cell migration (C), that were detected as significantly related biological processes in GO analysis, selected among those more than 2 fold down-regulated or 2 fold up-regulated genes in test sample vs control sample (Tables S1 and S2). Data are presented as mean ± SDs of n=3 samples. P value for the student-t test is shown in each graph. ∗*p* < 0.05, ∗∗*p* < 0.01, ∗∗∗*p* < 0.001.

## Expected outcomes

### Formation of spheroid using SF126

SF126 can form the compact spherical spheroid by plating on the ULA plate for 3 days (Figure 1A). However, some cell lines may not be able to form the compact spherical spheroids (Figure 1B, Miapaca-2 cell line). Such cell line is not suitable for use in the assay to evaluate the dispersion level of cancer cell invasion from the spheroid, because embedding them in atelocollagen gel will cause the structure to break down even further and will not be able to see the invasion cells dispersing from the compact spherical spheroid.

### Image acquisition of the live-cell imaging and their analysis

Live cell imaging of the control sample (spheroids not embedded in atelocollagen gel) and the test sample (spheroids embedded in atelocollagen gel) can be acquired using the Incucyte Zoom Live-Cell Analysis System (Sartorius). The movie or time-lapse images generated from the phase-contrast images taken every hour (Methods video S1; movie for control spheroid, Methods video S2; movie for test spheroid, Figure 2A; the tiff images taken every 6 h from the movie), allow you to kinetically monitor spheroid changes. For example, you can see that some of the SF126 cells in the test sample move out of the spheroid and invade into the atelocollagen gel. In addition, you can use the Incucyte zoom spheroid analysis software to quantify the increased degree of dispersion of invading cells observed in the test sample (Figure 2B). If you don’t have such a spheroid analysis software, you can manually quantify the dispersion level of invaded cells using the ImageJ Fiji INSIDIA open-source macro (Figure 3). In this case, create the black-and-white “mask image” (the image, which was manually segmented the spheroid and the invaded cells through setting the threshold), and plot the graph of the “mask image” (Figure 5). By measuring the plot area using the “wand tool” (Figures 5A and 5C), you can quantify the dispersion level of the invaded cells from the spheroid observed in each image. For example, you can clarify the robust time course increase in the dispersion level of the invaded cells compared to that of the control sample (Figure 5E).

### Quickly collecting the high-quality total RNA from whole atelocollagen gels containing the spheroid and dispersed invaded cells

Comprehensive gene expression analysis such as GeneChip Clariom S Assay (Filgen), for example, requires at least 1 μg of total RNA at a concentration of greater than 100 ng/μL with high quality, such that the ratio of absorbance at 260 nm and 280 nm is approximately 2.0. Using the method shown in Figure 6, you can easily sample the whole atelocollagen gels containing the spheroid and dispersed invaded cells from the each well of the 2 or 3 ULA plates, and collect a sufficient amount of high-quality total RNA by dissolving them with RNeasy mini kit (QIAGEN) as shown in Figure 6I. By using this total RNA in the GeneChip Clariom S Assay, you can obtain the comprehensive transcript expression data of your samples (Tables S1 and S2), and be able to proceed such as GO analysis (Figure 7A) and further RT-qPCR validation studies (Figures 7B and 7C).

## Limitations

The total RNA extraction method presented can also be applied to various 3D culture samples that are similarly embedded in the atelocollagen gel. By adding the desired chemicals, such as inhibitors, into the atelocollagen gel while solidifying the gel, you can also screen for inhibitors that are effective in suppressing the growth of your 3D culture samples or reducing the motility of invading cells, with collecting their total RNA to further proceed with comprehensive gene expression analysis. However, because this method collects the entire cells embedded in the atelocollagen gel to extract total RNA, only the bulk sample RNA can be extracted. For example, when using organoids composed of several different cell types, it would be limited to studying the effects of chemicals on each cell type that makes up the 3D samples.

## Troubleshooting

### Problem 1

Air bubbles form while mixing the 0.4% atelocollagen gel (related to step 2).

### Potential solution

0.4% atelocollagen solution is a highly viscous solution that easily contains air bubbles while mixing the solution. Pipetting the highly viscous solution with a high speed, smaller amount of solution will be aspirated by the pipette due to their high viscosity, which cause air to be expelled together into the solution while ejecting solution. In addition, due to their high viscosity, the amount of the solution sticks to the inner wall of the tip will increase, which cause the air will be expelled before the solution is expelled, leading to form bubbles in the solution. Therefore, we highly recommend to slowly and carefully mixing the solution.

### Problem 2

Loss of the spheroids while aspirating the culture medium (related to step 3a).

### Potential solution

It is recommended that the aspirated medium is discarded into a sterile 10 mm dish. In case you accidentally aspirate the spheroid, you can still see it with the naked eye in the discarded medium in the sterile 10 mm dish, and be able to collect the spheroid from there and return it to the 96-well plate.

### Problem 3

The image failed the automatic spheroid segmentation (related to step 6c, 6d).

### Potential solution

Automatic spheroid segmentation using INSIDIA open-source macro works very well with spheroid images that have clear spheroid outlines, but it often unable to work for the spheroid with dispersed invasion cells. In that case, you can alternatively use the manual segmentation with setting the threshold by your own as shown in step 6e and 6f.

### Problem 4

Unsuccessful dissolving of the spheroids after vigorous shaking of the RLT buffer containing atelocollagen gel (related to step 9i).

### Potential solution

Shake the tube up and down vigorously for further 3-5 min to further dissolve the remaining spheroid. In case you still unable to dissolve the spheroid, centrifuge the lysate solution, transfer the sup (the lysate solution without undissolved spheroid debris) to a new 15 mL tube, and collect the spheroid debris into the 1.5 mL tube. Add 100 μL the RLT buffer to the spheroid debris and homogenize the spheroid using a 1.5 mL tube homogenizer Pestles to completely dissolve the spheroid, and return it to the lysate solution transferred in the 15 mL tube.

### Problem 5

Poor yield of total RNA from the spheroids (related to step 9 and step 10).

### Potential solution

Ensure that the spheroids completely dissolved during homogenization in RLT buffer as described in the solution to problem 4 (step 9i). In addition, we recommend using fewer number of the RNeasy Mini Spin Column to collect the RNA to reduce the loss of lysate remaining on each column (step 10c). It is also highly recommended to collect the eluted RNA solution from the first column and use it for the elution of the second column to obtain a higher concentration of total RNA (step 10o).

## Resource availability

### Lead contact

Mayumi Fujita, fujita.mayumi@qst.go.jp.

### Technical contact

Mayumi Fujita, fujita.mayumi@qst.go.jp.

### Materials availability

This protocol did not generate new unique reagents.

### Data and code availability

This protocol did not generate datasets or code.

## Acknowledgments

We are grateful to the crew of Koken Co., Ltd. for providing expertise discussions about handling the 3D Ready Atelocollagen that greatly assisted the protocol development. This work was supported in part by Fund for the Grant-in-Aid for Scientific Research (C) (grant no. 22K07786 to M.F.) from the 10.13039/501100000646Japan Society for the Promotion of Science. The funder had no role in designing the study, in collection, analysis, and interpretation of data, or in writing the manuscript.

## Author contributions

M.F. designed and developed the protocols and wrote the manuscript. K.I. worked on the part of the protocol development and technical description for collecting total RNA. T.S. prepared the 3D Ready Atelocollagen. H.H., S.K., and T.M. worked on the part of the protocol development, specifically for real-time imaging. J.K. and A.Y. helped to summarize table. All authors read and approved the final manuscript.

## Declaration of interests

The authors declare no competing interests.

## Supplemental information

Table S1. List of more than 2-fold down-regulated genes in test sample vs control sample, related to step 10

Table S2. List of more than 2-fold up-regulated genes in test sample vs control sample, related to step 10
